# A Comparison of Severely Injured Patients after Suicide Attempts and Violent Crimes—A Retrospective Study of a Level 1 Trauma Center

**DOI:** 10.3390/clinpract14040118

**Published:** 2024-07-26

**Authors:** Heinz-Lothar Meyer, Thomas Reck, Christina Polan, Bastian Mester, Manuel Burggraf, Christian Waydhas, Sonja Vonderhagen, Marcel Dudda

**Affiliations:** 1 Department of Trauma, Hand and Reconstructive Surgery, University Hospital Essen, 45147 Essen, Germany; thomas.reck@uk-essen.de (T.R.); christina.polan@uk-essen.de (C.P.); bastian.mester@uk-essen.de (B.M.); christian.waydhas@uk-essen.de (C.W.); sonja.vonderhagen@uk-essen.de (S.V.); marcel.dudda@uk-essen.de (M.D.); 2 Department of Orthopaedics and Trauma Surgery, GFO Kliniken Mettmann-Süd, 40724 Hilden, Germany; manuel.burggraf@gfo-kliniken-mettmann-sued.de; 3 Department of Orthopaedics and Trauma Surgery, BG Klinikum Duisburg, 47249 Duisburg, Germany

**Keywords:** suicide, violent crime, violent offenses, rehabilitation after trauma, pre-existing psychiatric conditions and trauma, healthcare system, emergency room

## Abstract

Background: Seriously injured persons with pre-existing psychiatric conditions or those injured due to violent crimes represent a particularly vulnerable treatment group. Methods: All patients with injuries from suicidal attempts (PSAs) or patients with injuries from violent offenses (PVOs) that presented to the university emergency room of a Level 1 trauma center in Germany between 1 January 2017 and 31 November 2022 were retrospectively investigated. Results: It can be seen that PVOs were significantly younger compared to PSAs (*p* = 0.03). Total hospital stay was significantly longer for PSAs compared to PVOs (*p* < 0.001). PSAs were also significantly more severely injured than PVOs (*p* < 0.001). Our study was able to show a significant difference between both patient groups in the region of injury (*p* < 0.001). PSAs had a significantly more extensive psychiatric history than PVOs (*p* < 0.001). Conclusion: Injuries from suicide attempts and violent offenses are a serious and growing public health problem, but one that can be addressed through timely, evidence-based, and often cost-effective interventions. It requires early interaction among multiple disciplines and a standardized approach.

## 1. Introduction

Polytrauma refers to multiple, simultaneous injuries to different areas of the body sustained by a person, one or combination of which may be life-threatening. The injuries are often due to a significant event such as accidents or acts of violence [1]. Traumatic injuries are a leading cause of death and permanent damage to people worldwide. Worldwide, nearly 10% of all deaths are due to trauma [2]. The World Health Organization (WHO) reports that one in every eight people in the world live with a mental disorder and have known risk factors for increased mortality [3,4,5]. Worldwide, more than 700,000 people die by suicide each year. Suicide is the fourth leading cause of death among 15–29 year olds [6]. In Europe, 5% of patients hospitalized with major traumas are found to have a history or cause of suicide attempts [7]. This assumes that patients who have attempted suicide have more severe injuries than severely injured patients without a psychiatric history [8]. In Germany, 144,663 offenses of dangerous and grievous bodily harm were registered in 2022, according to police crime statistics from the Federal Criminal Police Office [9]. In Germany, crimes of violent crime and dangerous and grievous bodily harm increased by 19.8% and 18.2%, respectively, in 2022 compared with the previous year [10]. This shows that there are increasingly larger patient collectives of severely injured patients after suicide attempts (PSAs) and after violent offenses (PVOs). These patient groups represent a particularly vulnerable group who may suffer both physical and psychological impairment. As they progress, this patient group faces physical, emotional, and psychological challenges that can have profound effects on their recovery. These particular patient populations are often not treated holistically. In particular, follow-up treatment and early involvement of the departments of psychiatry, psychosomatics, and psychology often do not proceed optimally in everyday clinical practice. Severely injured patients with psychiatric disorders such as suicidality or patients severely injured due to violent crimes require comprehensive and multidisciplinary care.

In summary, both patient populations have not yet been well studied, have increasing patient numbers, and are very likely to have pre-existing psychiatric conditions. As a result, similar problems are assumed in inpatient and in post-hospital stays. The treatment of PSAs and PVOs is a complex matter that requires specialized knowledge, sensitivity, and therapies. The present study examines both patient collectives and compares them with each other in order to gain a better understanding of these patient collectives, to be able to treat them more effectively, and to recognize possible differences. This could lead to an improvement in patients’ medical and economic situation and, especially, post-hospital follow-up treatments after acute hospital stay.

## 2. Materials and Methods

### 2.1. Patient Collective

At the Department of Trauma, Hand and Reconstructive Surgery at Essen University Hospital, all patients with injuries involving a suicide attempt (PSAs) or patients with injuries due to violent crimes (PVOs) who presented to the emergency room and/or our intensive care unit in Germany between 1 January 2017 and 31 November 2022 were retrospectively studied and the patient groups were compared.

### 2.2. Characteristics

The data collected (sex, age, place of residence, date of admission, alert, admission by ambulance (RTW)/emergency medical service (EMT)/rescue helicopter (RTH)/autonomous, emergency room, ISS (“injury severity score”), secondary diagnoses, type of hospital admission, surgery, hospital stay, psychiatric presentation, type of suicide attempt, type of extraneous aggression, follow-up care) were part of routine clinical practice. Time of day was defined as 06:01 a.m.–12:00 a.m., 12:01 a.m.–6:00 p.m., 18:01 p.m.–24:00 p.m., and 00:01 a.m.–06:00 a.m. Based on the American Society of Anesthesiologists (ASA) classification, study participants’ pre-existing conditions were categorized. ASA class I corresponds to no pre-existing conditions in the present study. ASA class II corresponds here to pre-existing conditions with no or minor limitations in daily life, and ASA classification III corresponds to pre-existing conditions with severe limitations in daily life [11]. To assess the severity of the injuries, we determined the injury severity score (ISS) for each patient [12].

### 2.3. Statistical Analysis

Statistical analysis was performed using IBM SPSS Statistics (Version 29) software (IBM, Armonk, NY, USA). Descriptive data were analyzed for mean, standard deviation (M ± SD), median, and interquartile range (M (IQR)). All values were tested for normal distribution using the Kolmogorov–Smirnov test. Depending on whether a normal distribution was present, the *t*-test or Mann–Whitney U test was performed. The association of two categorical variables between independent samples was examined using a chi-square test. We considered values of *p* < 0.05 significant and those of *p* < 0.001 highly significant.

### 2.4. Objective of Study

PSAs and PVOs have not yet been well studied. Due to the increasing number of cases, the aim of this study is to register, examine, and compare both patient collectives. This will contribute to a better understanding of both patient populations and, on the basis of this and further studies, may enable more effective treatments in the future.

## 3. Results

In the period from 1 January 2017 to 31 November 2022, 126 patients after suicide attempts and 126 patients after violent crimes were registered through the emergency room or/and admitted to our ICU. The results and exact distributions are shown in Table 1.

### 3.1. Seriously Injured Patients after Suicide Attempts

Of the 126 PSAs, 70.6% (89) were male and 29.4% (37) were female. More males than females died by suicide, showing no significant difference (*p* = 0.092). The overall mean age of the injured was 47.11 (±20) years; for males, it was 47.3 (±21.3) years, and for females, it was 46.6 (±17.1) years. Most of the PSAs (85.7% (108)) resided in large cities. The most common accident location was also in large cities (90.5% (114)). The place of residence and accident location were the same in 85.7% (108) of PSAs. The rescue service was called most frequently by neighbors or passers-by in the majority of cases (19% (24)). A total of 96.8% (122) of PSAs were taken to the hospital by an ambulance (RTW) and emergency doctor. The most common time of day of hospital admission was the afternoon (33.3% (42)). The type of suicide was jumping from a great height (>3 m) in 45.2% (57) of cases, attempted suicide with a knife in 32.5% (41), jumping in front of a train or car in 11.9% (15), the use of a firearm in 5.6% (7), and hanging in 4% (5). The length of hospital stay in this patient population was 27.23 (±52.93) days, including an average of 11.87 (±16.1) days in the intensive care unit (ICU). The ISS was over 16 for 51.6% (65) of patients and averaged at 21.17 (±20.8) overall. During hospital stay, 13.5% (17) of the patient group died and this occurred after an average of 12.88 (±17.23) days. Surgery had to be performed in 79.4% (100) of PSAs in our hospital. In 48.4% (61) of the patients, the injuries were in more than two different regions. The exact distribution is shown in Table 1. A total of 61.1% (77) of PSAs had previously diagnosed psychiatric disorders. After hospital admission, 16.7% (21) of the patient group was estimated by the psychiatrist to be suicidal. National health insurance covered 79.4% (100) of the admitted patients. After hospital treatment in the acute hospital, 37.3% (47) of patients were discharged home, 34.9% (44) were transferred to psychiatric care, 6.3% (8) were transferred to geriatric care, 2.4% (3) each were discharged to short-term care (STC), early neuro-rehabilitation, or a correctional facility (JVA), and 0.8% (1) were discharged to long-term care (LTC).

### 3.2. Severely Injured Patients after Violent Crimes

Of the 126 PVOs, 80.2% (101) were male and 19.8% (25) were female. More males than females were injured by violence, showing no significant difference (*p* = 0.092). The overall mean age of the injured was 39.81 (±18.76) years, 37.8 (±17.4) years for males, and 48.2 (±21.9) years for females. Most of the injured patients lived in large cities (92.9% (117)). The most common place of accident was in large cities (96% (121)). The place of residence and accident location were the same in 84.9% (107) of injured patients. The rescue service was notified most frequently by neighbors or passers-by (15.9% (20)). A total of 86.5% (109) of the injured patients were brought to the hospital by RTW and an emergency doctor. The most common time of day of hospital admission was the evening (42.9% (54)). The type of aggression was assault with a knife in 45.2% (57) of cases, blunt force without an object in 33.3% (42), blunt force with an object in 18.3% (23), and the use of a firearm in 3.2% (4). The length of hospital stay in this patient population was 7.1 (±11.36) days, of which an average of 3.8 (±7.1) days were spent in the ICU. The ISS was over 16 for 35.7% (45) of patients and averaged at 11.7 (±14.7). During hospital stay, 4.8% (6) of the patient group died and this occurred after an average of 12.7 (±15.5) days. Surgery had to be performed on 41.3% (52) of the injured patients in our hospital. The most frequently injured region was the head/spine region (48.4% (61)). The exact distribution is shown in Table 1. A total of 19.8% (25) of injured patients had prediagnosed psychiatric conditions. National health insurance covered 81.7% (103) of admitted patients. After hospital treatment in the acute hospital, 84.9% (107) of patients were discharged home, 5.6% (7) were transferred to early neuro-rehabilitation, 1.46% (2) were discharged to psychiatric or correctional facilities, and 0.8% (1) were discharged to geriatric care or LTC.

### 3.3. Comparison of Groups of Severely Injured Patients after Suicide Attempts and after Violent Crimes

It can be seen that PVOs were significantly younger compared to PSAs (*p* = 0.03). Total hospital stay was significantly longer in the PSA group than in the PVO group (*p* < 0.001). ICU stay was also significantly longer in the suicidal patient population (*p* < 0.001). The ISS was significantly higher in the PSA group (*p* < 0.001). There is a significant difference between the two groups in the time of day of presentation to the ED (*p* = 0.005) but no significant difference in the time of year. In our study, no significant association was seen between the groups of PSAs or PVOs and gender (*p* = 0.092). A significant association of both groups was seen with admission via the emergency room (*p* < 0.001). PSAs required surgery significantly more often than PVOs (*p* < 0.001). Similarly, there is a significant difference between both patient groups in the region of injury (*p* < 0.001). Suicidal severely injured patients had a significantly more extensive psychiatric history than patients severely injured by violent crimes (*p* < 0.001). There is a significant difference in follow-up care between both patient groups (*p* < 0.001).

## 4. Discussion

In our study, we observed that both in the patient group of suicidal attempts (PSA) and patients severely injured by violent offenses (PVOs), the majority were men. This can be confirmed by other studies. Petrosky et al. also observed more males than females involved in suicide attempts in the United States in 2017 [13]. In contrast, Piazzalunga et al. reported a majority of severely injured women due to suicide attempts by jumping between 2014 and 2016 in the Bergamo area of Italy in their study [14]. Ajayi et al. recorded predominantly males (85.6%) with stranger-inflicted aggressive injuries from a knife in London between 2014 and 2018 [15]. Also, in our study, predominantly men (80.2%) were seriously injured by violent offenses. Pino et al. also observed predominantly men with penetrative injuries due to violent acts [16]. In our study, the overall mean age of those injured was 39.81 (±18.76) years. The mean age of those injured by suicide was 47.11 (±20); those injured by violent crimes were significantly younger at 39.81 (±18.76) years (*p* = 0.03). Nohl et al. also observed a similar mean age of severely injured patients after a suicide attempt of 42.3 years in their study and reported a younger age of injured patients after a suicide attempt compared to trauma patients in general [8]. In Persett et al.’s study, the median age of patients after a suicide attempt was also 42 years [17]. Other studies reported a younger median age of those seriously injured by violent acts at 16–25 years (Ajayi et al.) or 29 years (Pino et al.) [15,16]. The young average age may be related to their own experiences of violence at a young age, the increasing influence of violence in the media, and the general pressure and stressful situations in young adulthood. The vast majority of suicidally polytraumatized patients resided in large cities. A similar distribution was seen among patients injured by violent acts (large cities). In both patient groups, the place of residence and the place of accident were identical in approximately 85% of cases. To date, there is a paucity of studies in this regard. Coles et al. observed injuries caused by violent crimes predominantly in cities rather than in rural areas [18]. This is probably due to the higher population density in large cities.

The most frequent time of day of admission to the hospital for PSAs was the afternoon. In contrast, the most frequent time of day of admission for PVOs was the evening. This is also shown by the study of Ajayi et al. in which most injuries caused by violent crimes with a knife in London in the years between 2014 and 2018 were observed on Saturdays between 22:00 and 0:00 [15]. This may be due to the fact that many people, especially young people, gather in the evening and attacks can happen under the cover of darkness. In our study, no season can be identified in which significantly more suicidal attempts or violent crimes are registered. This reflects the current literature.

In the study we conducted, the most common type of attempted suicide was jumping from a great height (>3 m) followed by attempted suicide with a knife. The most common type of aggression against PVOs was assault with a knife. The current literature also shows a significant increase in violent offenses involving a knife [15]. Pino et al. studied injured persons according to violent offenses in the Boston area (USA) and recorded firearm injuries most frequently, with an increased number of cases during the COVID-19 pandemic [16]. A study by Bieler et al. examined data from the German Trauma Registry between 2009 and 2018 and indicated that gunshot and stab wounds have a low incidence in Germany by comparison and are mostly caused by violent crimes or suicide attempts. We were able to confirm these severe injuries in our study [19]. Jumping from a great height is well studied in the literature and is often reported as a suicide attempt. Piazzalunga et al. reported jumping from a great height as the most common method of suicidal attempts in northern Italy [14]. In their study, Persett et al. most frequently observed cutting injuries (34%) followed by jumping from a great height (32%) as a suicide attempt [17]. Petrosky et al. registered the use of a firearm as the most common suicide attempt among men and poisoning among women in 2017 in the United States, which is certainly related to national laws [13]. The WHO states that pesticide ingestion, hanging, and firearms are the most common methods of suicide in the world [6]. These types of suicide and violent crimes can probably be observed most frequently, as they are easier to overcome than the other types and are easier to obtain or implement.

The length of hospital stay was significantly longer for PSAs than for PVOs (*p* < 0.001). ICU stay was also significantly longer for PSAs (*p* < 0.001). This is probably related to the overall higher ISS for PSAs. Piazzalunga et al. reported an ICU admission rate of 57.5% for suicidally injured patients in their study, with a mean length of stay of 26 (±24.34) days. The length of stay is similar to the data we registered for PSAs [14]. Nohl et al. reported the stay in the ICU to be 17 days on average [8]. Persett et al. observed a stay of 14.3 days for PSAs in his study [17]. Omi et al. also recorded a prolonged stay for PSAs [20]. Pallett et al. registered a stay of 3.04 days for PVOs, which is shown to be lower than in our study [21].

The average ISS was 21.17 (±20.8) for PSAs. In their study, Piazzalunga et al. reported a similar ISS in suicidal jumpers of 18.9 (±11.8) [14]. Nohl et al. reported the ISS for PSAs to average at 24.7 [8]. The average ISS was 11.7 (±14.7) for PVOs. The ISS was significantly higher for PSAs than for PVOs (*p* < 0.001). During hospital stay, 13.5% (17) of the PSAs died and this occurred on average after 12.88 (±17.23) days. During hospital stay, 4.8% (6) of PVOs died and this occurred on average after 12.7 (±15.5) days. Piazzalunga et al. reported a lower mortality of 7.5% for PSAs in their study, while Nohl et al. reported a slightly higher mortality of 18% for PSAs [8,14]. PSAs required surgery significantly more often than PVOs in our clinic (*p* < 0.001). Piazzalunga et al. reported a similar number of surgical interventions needed for PSAs (75%) [14]. Ajayi et al. stated that 14% of PVOs needed surgical care [15]. This may be due to the fact that PSAs want to die by suicide, whereas the offenders who injure PVOs usually focus on injuring another person.

In 48.4% (61) of PSAs, the injuries were in more than two different regions in our study and the most frequently affected body region was the trunk. Most injuries among PVOs were commonly found in the head/spine region. Similarly, there is a significant difference between both patient groups in the region of injury (*p* < 0.001). Current studies can confirm this. Piazzalunga et al. reported injuries to the face, abdomen, and extremities more frequently for PSAs in their study [14]. Nohl et al. stated that a suicide attempt often correlates with polytrauma [8]. Dekker et al. reported head and neck injuries most frequently for PSAs in their study [22]. Omi et al. and Wirbel et al. observed injuries to the lower extremities and spine most frequently [20,23]. Ajayi et al. observed predominantly thoracic (39%) and abdominal (26%) injuries following xenophobic behavior [15]. Pallett et al. also observed thoracic and abdominal injuries most frequently for PVOs [21].

PSAs had significantly more pre-existing psychiatric conditions than PVOs in our study (*p* < 0.001). This results from the connection between psychiatric disorders, such as depression, and suicide [24]. In their study, Nohl et al. stated that prolonged hospital stay and mortality might be related to pre-existing psychiatric conditions and involve a lack of cooperativeness. Here, depression and schizophrenia were reported as the most common pre-existing psychiatric conditions [8]. Omi et al. and Nielssen et al. also most frequently recorded schizophrenia as a pre-existing condition before suicidal attempts [20,25]. Coles et al. observed sequelae in PVOs such as post-traumatic stress disorder, further mental illnesses, or substance abuse [18,26]. Various studies have confirmed that patients with psychiatric disorders have a higher risk of surgical complications, an increased risk of post-traumatic mortality, and a prolonged hospital stay. Decker et al. report an almost doubled risk of complications [22]. This illustrates that these patients require special somatic and psychiatric therapy [27]. This must be considered during treatment in an acute hospital and especially in follow-up care in order to develop adequate standardized therapy. Multimodal therapy between several specialist departments is necessary from the beginning of the acute phase in an acute hospital, considering the somatic and psychological needs of the patients [28]. This should be achieved using a standardized algorithm. Fischer et al. observed in their study that PVOs also had a high number of physical and psychological trauma events in their history [29]. Nohl et al. stated that PSA discharges were often different. In their study, 56% were discharged to a psychiatric facility, only 45 were discharged home, and almost none were discharged to rehabilitative facilities [8]. This contrasts our study in which the majority of PSAs and PVOs were discharged home. Here, standardization should take place. Rehabilitation with more possibilities to address the different aspects of these two patient groups to avoid recurrence or sequelae is urgently needed. In particular, new centers and offers of rehabilitation must be created that unite musculoskeletal rehabilitation with psychiatric therapies [30,31]. This is already supported by other studies from the US, which are testing different care options after acute hospital stay [31]. The study by Hirot et al. found that a transdisciplinary unit with a dual approach of rehabilitation (physiotherapy, occupational therapy, etc.) and psychiatric care as therapy for seriously injured suicidal patients shows a reduction in acute hospital stay and can thus reduce the costs of the healthcare system for the patients [32]. A study by Jansen et al. also showed that psychiatric comorbidities lead to a prolonged acute hospital stay, an increased rate of rehospitalization, and thus higher medical costs [33]. Furthermore, close collaboration during acute hospital stay between somatic and psychiatric therapies is already required. These show a high potential of cost savings for the healthcare system.

### Limitations of This Study

Both patient groups have suffered different traumas and had different injuries in different body regions. Similarly, no distinction was made between individual psychiatric comorbidities. Only patients who survived a suicide attempt or a violent crime and made it to the hospital could be included in our study. There may be distortions in the gender distribution here, particularly in the PSA group, as it has been established in the literature, for example, that women attempt suicide more frequently and men complete it more often [13,34,35]. Patients under the age of 18 were not included in this study. Nevertheless, the findings remain valid and justify the search for an improvement in the medical and economic situation and, especially, post-hospital follow-up treatments after an acute hospital stay for these two complex patient groups.

## 5. Conclusions

Severe injury from suicide attempts, and gun violence in particular, is a serious and growing public health problem, but one that can be addressed through timely, evidence-based, and often cost-effective interventions. The treatment of PSAs and PVOs is very complex and requires special knowledge and sensitivity. It is not rare for physical trauma to be accompanied by psychological trauma, and not only in patients with pre-existing conditions. PSAs and PVOs are often unable to correctly adhere to or follow the treatment recommendations of the individual specialist disciplines due to their pre-existing psychiatric conditions, which can lead to a poorer outcome and a longer hospital stay. The necessary psychiatric or psychosomatic–psychotherapeutic treatment often must be delayed as long as somatic consequences of the injury are still present, as this specialty is often not represented in German acute hospitals. Of course, life-saving emergency procedures are the first priority. Suicidal tendencies may still be present, leading to increased care requirements and patient monitoring. Once again, drug sedation or restraint may be necessary, with the consequences mentioned above. Post-inpatient follow-up treatment is also often a problem. Transfer to a psychiatric or psychosomatic specialist hospital is often not possible, as care for complex somatic restrictions such as immobility, dressing changes, etc., is generally not guaranteed here. This means that there is a lack of suitable care facilities that combine these aspects [8]. This can also lead to prolonged hospital stays and poorer patient outcomes. In our view, prophylaxis and the early involvement of psychosomatic–psychotherapeutic specialists in acute hospitals play a key role here. Patients could be continuously monitored regarding the development of symptoms of psychological stress. Regular early interdisciplinary care for polytrauma patients with and without pre-existing mental illnesses, both in the acute phase and in the further course of treatment, would be forward-looking. Early diagnosis with the help of personal and digital screening procedures for mental health, the provision of low-barrier discussions and eHealth services, and accompanying medication could be an initial approach to providing better care for patients still requiring intensive care after polytrauma. This promotes adherence, and mental health crises can be detected early and perhaps even prevented. Networks and close cooperation are essential for the realization of such a project. If the psychiatric–psychosomatic care of mentally ill polytrauma patients remains in one hand from the intensive care unit to outpatient post-inpatient treatment, long-term support and stabilization can take place. This could lead to an improvement in the medical and economic situation and, above all, the post-hospital follow-up treatment of both patient groups and reduce the high recurrence and complication rates. For national measures to be effective, a comprehensive, cross-sectoral prevention strategy is required [6]. There is certainly still a need for action here, particularly in Germany.

- An increasing number of polytrauma patients have psychiatric comorbidities that can significantly reduce their outcomes.
- The intensive care of polytrauma patients with pre-existing mental illnesses or acute psychological stress can often be prolonged or complicated.
- Polytraumatized patients who have attempted suicide or suffered violence offenses require close interdisciplinary cooperation between somatic and psychiatric/psychosomatic disciplines in order to ensure the best possible care from the emergency room to the rehabilitation clinic.
- Continuous interdisciplinary somatic/psychiatric/psychosomatic care within the context of established network structures from the intensive care unit to rehabilitation should be aimed for and can significantly improve patient outcomes and minimize costs.
- There is certainly still a need for improvement, especially in Germany.

## Figures and Tables

**Table 1:** All patients involving injuries from suicidal attempts (PSAs) or patients with injuries from violent offenses (PVOs) that were presented to the university emergency room of a Level 1 trauma center between 1 January 2017 and 31 November 2022 were prospectively investigated.

		Suicidal Attempts (PSAs) Number (n); Relative Frequency (%)	Violent Offenses (PVOs) Number (n); Relative Frequency (%)
gender	male	89; 70.6%	101; 80.2%
	female	37; 29.4%	25; 19.8%
residence	big city	108; 85.7%	117; 92.9%
	mid-sized city	14; 11.1%	4; 3.2%
	small town	2; 1.6%	4; 3.2%
	homeless	2; 1.6%	1; 0.8%
accident location	big city	114; 90.5%	121; 96%
	mid-sized city	10; 7.9%	3; 2.4%
	small town	0; 0	0; 0
	country cities	1; 0.8%	0; 0
	homeless	1; 0.8%	0; 0
call for rescue service	neighbors/passers-by	24; 19%	20; 15.9%
	relatives	11; 8.7%	3; 2.4%
	police/fire brigade	0; 0	9; 7.1%
	own alarm	0; 0	4; 3.2%
	unknown	0; 0	90; 71.4%
means of transport	rescue helicopter	3; 2.4%	2; 1.6%
	emergency doctor	122; 96.8%	109; 86.5%
	ambulance	1; 0.8%	7; 5.6%
	patient transporter	0; 0	0; 0
	autonomous	0; 0	7; 5.6%
	police	0; 0	1; 0.8%
time of day of admission to hospital	morning	27; 21.4%	20; 15.9%
	afternoon	42; 33.3%	22; 17.5%
	evening	39; 31%	54; 42.9%
	night	18; 14.3%	30; 23.8%
season of accident	spring	33; 26.2%	32; 25.4%
	summer	35; 27.8%	31; 24.6%
	autumn	40; 31.7%	37; 29.4%
	winter	18; 14.3%	26; 20.6%
injury level	emergency room	124; 98.4%	110; 87.3%
	ISS ≥ 16	65; 51.6%	45; 35.7%
	deceased	17; 13.5%	6; 4.8%
	surgical treatment	100; 79.4%	52; 41.3%
type of injury	jump from great height (>3 m)	57; 45.2%	0; 0
	knife	41; 32.5%	57; 45.2%
	jump in front of a train/car	15; 11.9%	0; 0
	firearm	7; 5.6%	4; 3.2%
	hanging	5; 4%	0; 0
	blunt violence without object	0; 0	43; 33.3%
	blunt violence with object	0; 0	23; 18.3%
region of injury	≥2 different regions	61; 48.4%	14; 11.1%
	exclusively torso	25; 19.8%	39; 31%
	exclusively head/spine	18; 14.3%	61; 48.4%
	exclusively upper extremities	12; 9.5%	9; 7.1%
	exclusively lower extremities	9; 7.1%	3; 2.4%
Pre-existing conditions	ASA class 1	42; 33.3%	85; 67.5%
	ASA class 2	77; 61.1%	40; 31.7%
	ASA class 3	7; 5.6%	1; 0.8%
	pre-existing psychiatric conditions	77; 61.1%	25; 19.8%
suicidality (according to psychiatric presentation)	still suicidal	21; 16.7%	--
	no longer suicidal	105; 83.3%	--
post-hospital care	home care	47; 37.3%	107; 84.9%
	psychiatry	44; 34.9%	2; 1.6%
	geriatrics	8; 6.3%	1; 0.8%
	STC (short-term care)	3; 2.4%	0; 0
	LTC (long-term care)	1; 0.8%	1; 0.8%
	early neuro-rehabilitation	3; 2.4%	7; 5.6%
	JVA (correctional facility)	3; 2.4%	2; 1.6%

## Data Availability

The raw data supporting the conclusions of this article will be made available by the authors on request.
